# Supplementary material for: Multiple Instances of Adaptive Evolution in Aquaporins of Amphibious Fishes
Source: Biology (Basel). 2023 Jun 12;12(6):846. doi: 10.3390/biology12060846 (PMC10295795; doi:10.3390/biology12060846)
Supplement: Supplementary file 1 [file biology-12-00846-s001.zip › Captions of Supplementary Figures.pdf]

Multiple instances of adaptive evolution in aquaporins of amphibious fishes

Lorente-Martínez H., Agorreta A., Irisarri I., Zardoya R., Edwards S.V., San Mauro D.

Captions of Supplementary Figures

Figure S1. Maximum likelihood (IQ-TREE) phylogram of vertebrate AQPs based on 441 aligned amino acid positions. Aquaporin main four groups are indicated with colour lines (blue for aquaglyceroporins, orange for superaquaporins, green for aquaammoniaporins and yellow for water-selective classical aquaporins). Aquaporin paralog classes are named (AQP0-16) and paralog groupings within each class are denoted by letters (a, b, a1, a2) on the corresponding branches. Black bullets on nodes denote support for Ultrafast Boostrap (UFBoot) and SH-aLRT (as implemented in IQTree) and rapid bootstrap (as implemented in RAxML). Half-blue bullets denote support for UFBoot and SH-aLRT only. Half-grey bullets denote support for rapid bootstrap only.

Figure S2. Results of Bayesian analyses of the AQP gene family across 62 vertebrate species. (A) The guide phylogeny used to analyze gene family evolution was obtained from the Timetree of Life database (www.timetree.org). Red branches indicate cases of gene expansion, orange branches cases of gene constriction, and blue branches cases of gene repertoire maintenance. Bars on the right indicate the number of AQP copies present in the studied species.

Figure S3. Sequence alignment of the AQP11b HH1 NPA motif region of Gobioidei and Apogonidae, including data from species whose genome sequence has been recently made available (indicated in bold type). For each sequence, the GenBank accession number is indicated in parenthesis.

Figure S4. 3D structural model reconstructions of some relevant AQP genes. Superimposition of the AlphaFold2 model (blue) with human AQP1 (PDB ID: 1H6I) (beige). (**A**) *Periophthalmus magnuspinnatus* AQP11b; (**B**) *Neogobious melanostomus* AQP11b; (**C**) *Danio rerio* AQP11a; (**D**) *Sphaeramia orbicularis* (**E**) *Danio rerio* AQP1a; and (**F**) RefSeq human AQP1.

Figure S5. Quality of the structures of Fig. S4 based upon the pLDDT parameter. (**A**) *Periophthalmus magnuspinnatus* AQP11b; (**B**) *Neogobious melanostomus* AQP11b; (**C**) *Danio rerio* AQP11a; (**D**) *Sphaeramia orbicularis* (**E**) *Danio rerio* AQP1a; and (**F**) RefSeq human AQP1.

Figure S6. Superimposition of the AlphaFold2 modelled aquaporins from Fig. S4. Beige: human AQP1 (PDB ID: 1H6I); Blue: *Periophthalmus magnuspinnatus* AQP11b; Pink: *Neogobious melanostomus* AQP11b; Green: *Danio rerio* AQP11b.; Red: *Sphaeramia orbicularis*.

Figure S7. Structural view of positively selected positions of the studied amphibious fish AQPs. NPA boxes (purple) and ar/R filter sites (orange). (**A**) AQP3 and AQP10 paralogs sites are mapped onto the three-dimensional structure of *Homo sapiens* AQP10 (PDB ID: 6F7H). Blue dots correspond to *Cyprinodon variegatus* AQP3b, green to *Clarias batrachus* AQP3b

branches, red to *Channa argus* AQP3b, and yellow to *Salarias fasciatus* AQP10b. (**B**) AQP8 paralog sites are mapped onto the three-dimensional structure of *H. sapiens* AQP1 (PDB ID: 1H6I). Blue dots correspond to *Parablennius parvicornis* AQP8a1 and green to *Monopterus albus* AQP8a1 branches. Black dots correspond to a shared position between *P. parvicornis* and *M. albus* AQP8a1 branches. (**C**) Sites of AQP11 paralog mapped onto the three-dimensional structure of *H. sapiens* AQP1 (PDB ID: 1H6I). Blue dots correspond to *M. albus* AQP11b, green to *Betta splendens* AQP11b, pink to *S. fasciatus* AQP11a, red to the Gobiidae clade AQP11b, and yellow to *Kryptolebias marmoratus* AQP11b. Black dots correspond to a shared position among *B. splendens*, *K. marmoratus* and the Gobiidae clade AQP11b branches.
