# Supplementary material for: Multiple Instances of Adaptive Evolution in Aquaporins of Amphibious Fishes
Source: Biology (Basel). 2023 Jun 12;12(6):846. doi: 10.3390/biology12060846 (PMC10295795; doi:10.3390/biology12060846)
Supplement: Supplementary file 1 [file biology-12-00846-s001.zip › Figure S3.pdf]

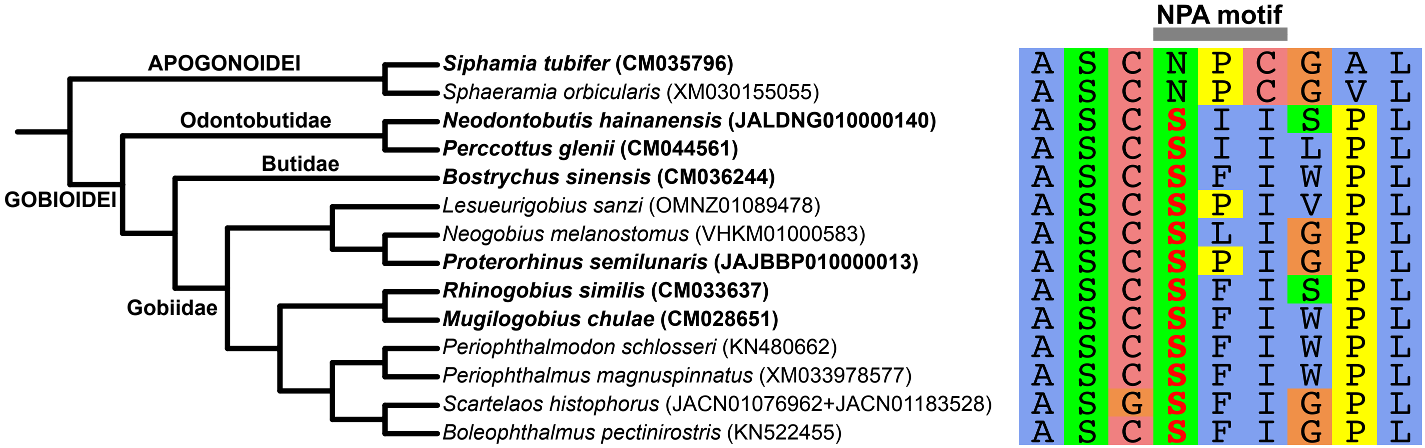
NPA motif
APOGONOIDEI
Odontobutidae
Butidae
GOBIOIDEI
Gobiidae
Siphamia tubifer (CM035796)
Sphaeramia orbicularis (XM030155055)
Neodontobutis hainanensis (JALDNG010000140)
Perccottus glenii (CM044561)
Bostrychus sinensis (CM036244)
Lesueurigobius sanzi (OMNZ01089478)
Neogobius melanostomus (VHKM01000583)
Proterorhinus semilunaris (JAJBBP010000013)
Rhinogobius similis (CM033637)
Mugilogobius chulae (CM028651)
Periophthalmodon schlosseri (KN480662)
Periophthalmus magnuspinnatus (XM033978577)
Scartelaos histophorus (JACN01076962+JACN01183528)
Boleophthalmus pectinirostris (KN522455)
A S C N P C G A L
A S C N P C G V L
A S C S I I S P L
A S C S I I L P L
A S C S F I W P L
A S C S P I V P L
A S C S L I G P L
A S C S P I G P L
A S C S F I S P L
A S C S F I W P L
A S C S F I W P L
A S C S F I W P L
A S G S F I G P L
A S C S F I G P L
