# Supplementary material for: Multiple Instances of Adaptive Evolution in Aquaporins of Amphibious Fishes
Source: Biology (Basel). 2023 Jun 12;12(6):846. doi: 10.3390/biology12060846 (PMC10295795; doi:10.3390/biology12060846)
Supplement: Supplementary file 1 [file biology-12-00846-s001.zip › Table S2.pdf]

Table S2. Results of the recombination analyses performed with the GARD software.

**Aquaporin**	**Recombination**
0	No recombination found
1	No recombination found
3	No recombination found
4	No recombination found
7	No recombination found
8	No recombination found
9	No recombination found
10	No recombination found
11	No recombination found
12	Recombination found
14	No recombination found
15	Recombination found
