# Supplementary figures and images for: Multiple Instances of Adaptive Evolution in Aquaporins of Amphibious Fishes
Source: Biology (Basel). 2023 Jun 12;12(6):846. doi: 10.3390/biology12060846 (PMC10295795; doi:10.3390/biology12060846)

# Duplication events

★ WGD

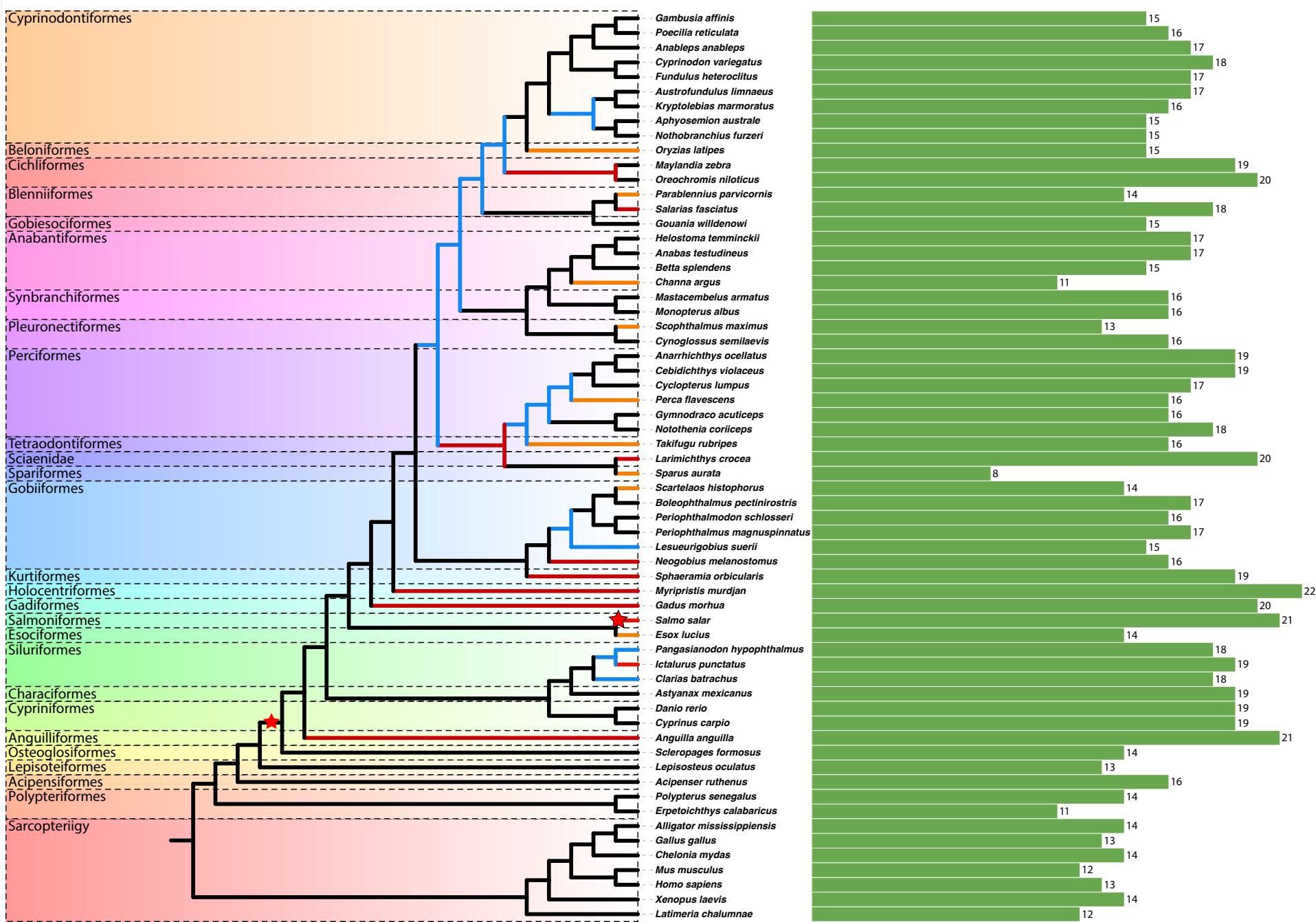

Supplement: Supplementary file 1 [file biology-12-00846-s001.zip › Figure S2.pdf]

A

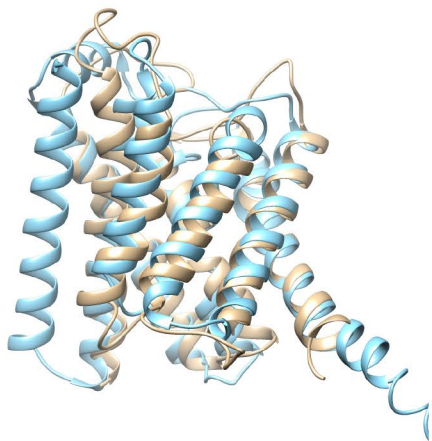

B

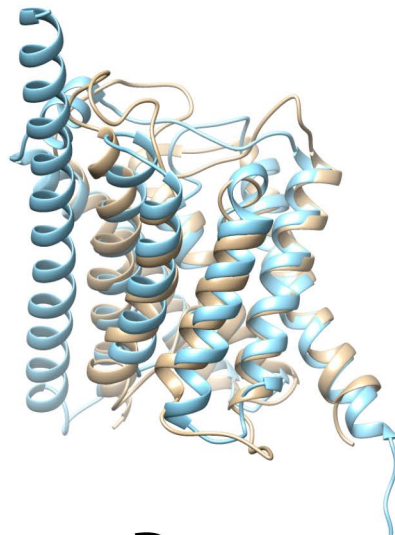

C

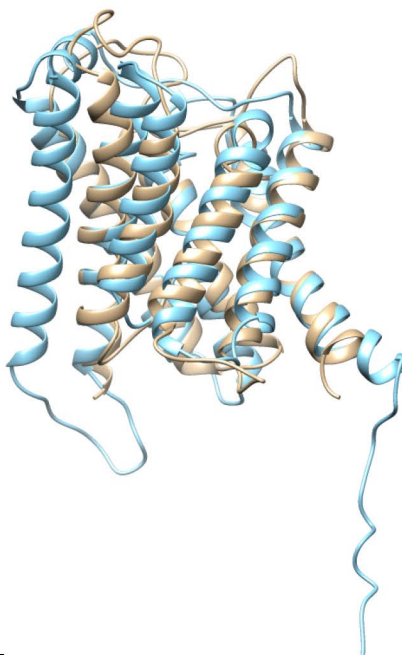

D

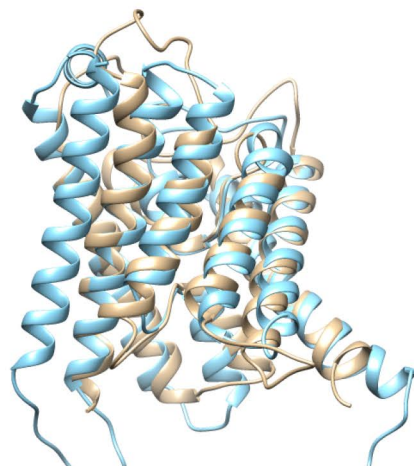

E

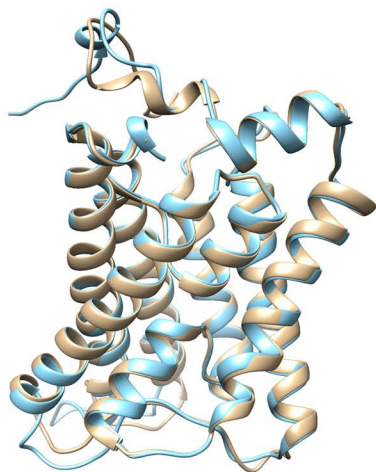

F

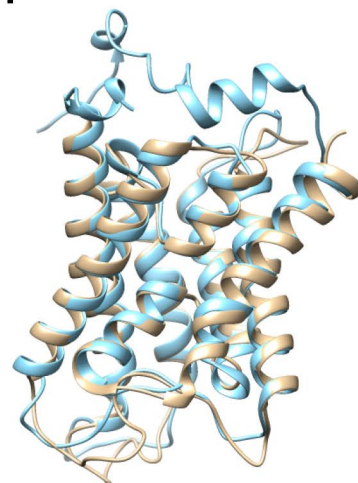

Supplement: Supplementary file 1 [file biology-12-00846-s001.zip › Figure S4.pdf]

A

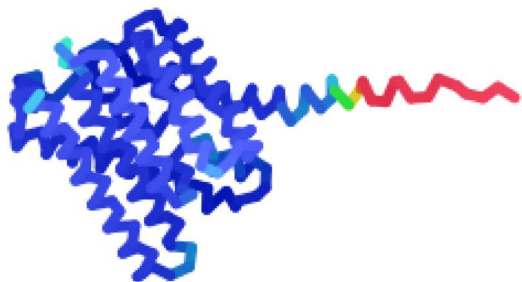

B

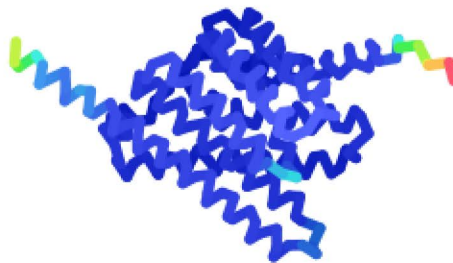

C

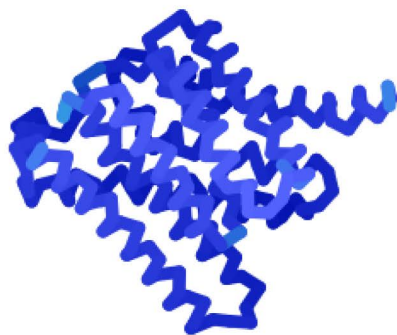

D

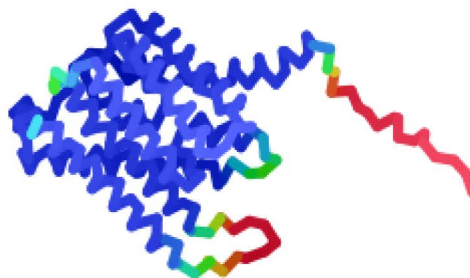

E

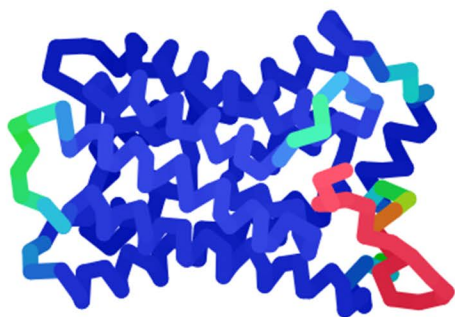

F

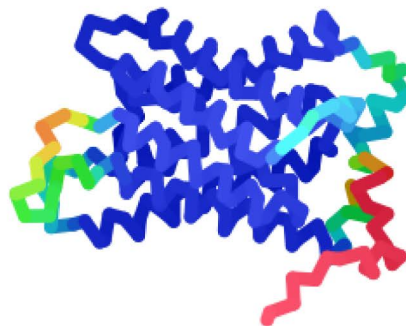

pLDDT: ■ Very low (<50) ■ Low (60) ■ OK (70) ■ Confident (80) ■ Very high (>90)

Supplement: Supplementary file 1 [file biology-12-00846-s001.zip › Figure S5.pdf]

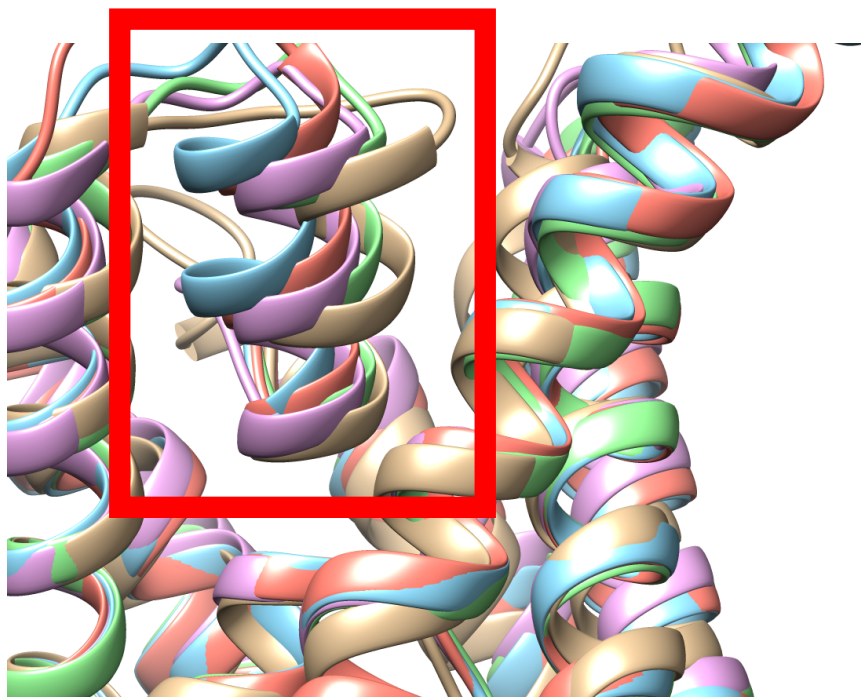

Supplement: Supplementary file 1 [file biology-12-00846-s001.zip › Figure S6.pdf]

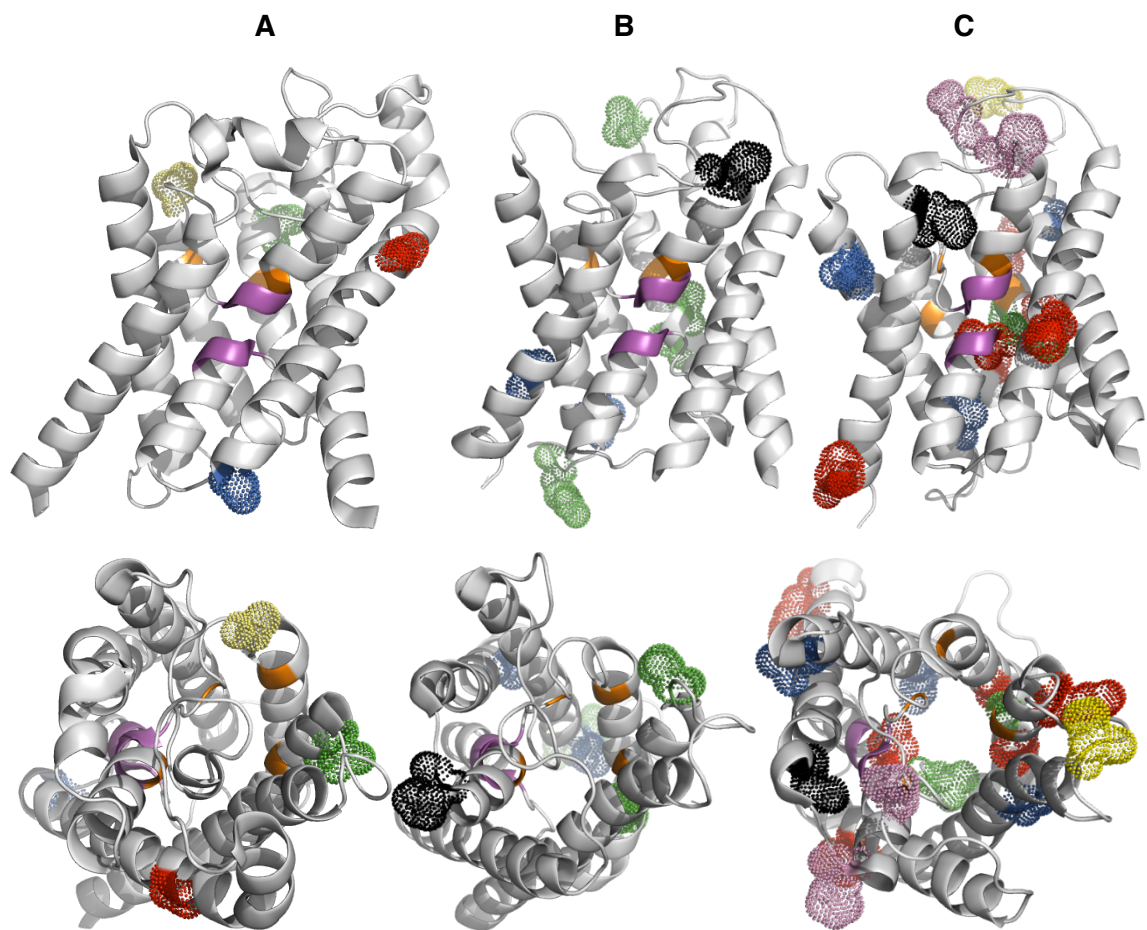

Supplement: Supplementary file 1 [file biology-12-00846-s001.zip › Figure S7.pdf]
