# Supplementary material for: Multiple Instances of Adaptive Evolution in Aquaporins of Amphibious Fishes
Source: Biology (Basel). 2023 Jun 12;12(6):846. doi: 10.3390/biology12060846 (PMC10295795; doi:10.3390/biology12060846)
Supplement: Supplementary file 1 [file Figure S1.pdf › Figure S1.pdf]

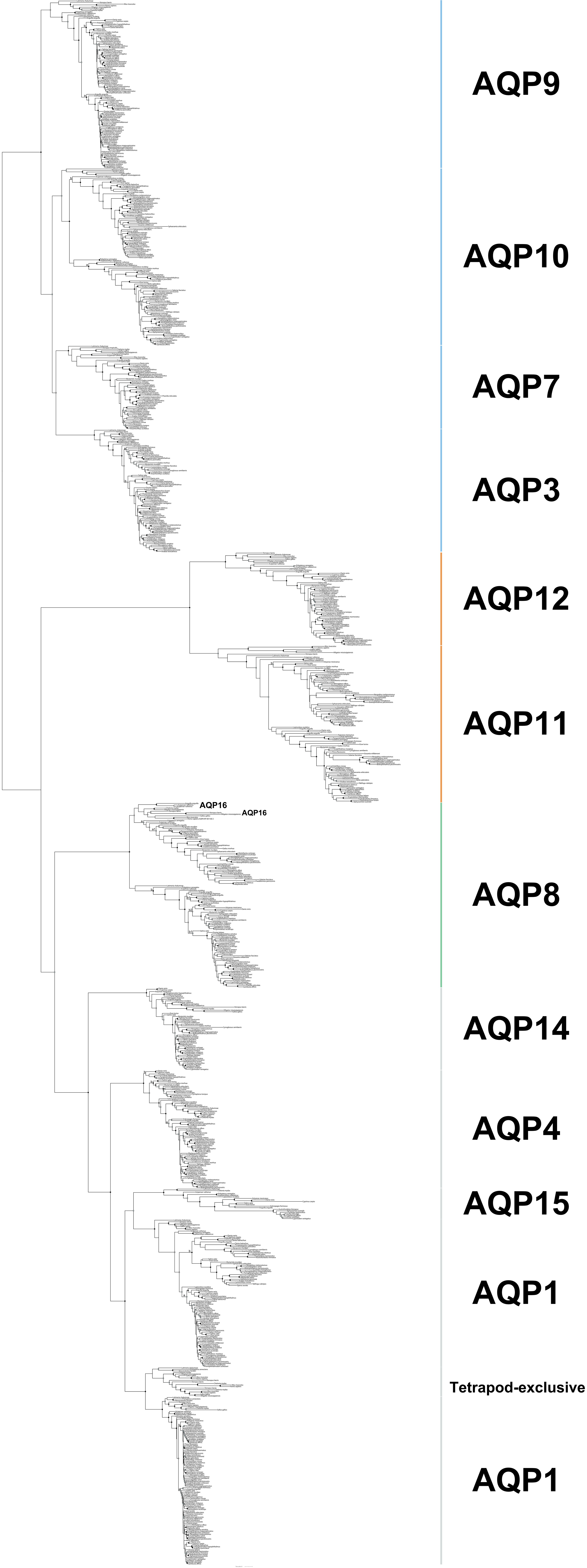
AQP9
AQP10
AQP7
AQP3
AQP12
AQP11
AQP16
AQP16
AQP8
AQP14
AQP4
AQP15
AQP1
Tetrapod-exclusive
AQP1
